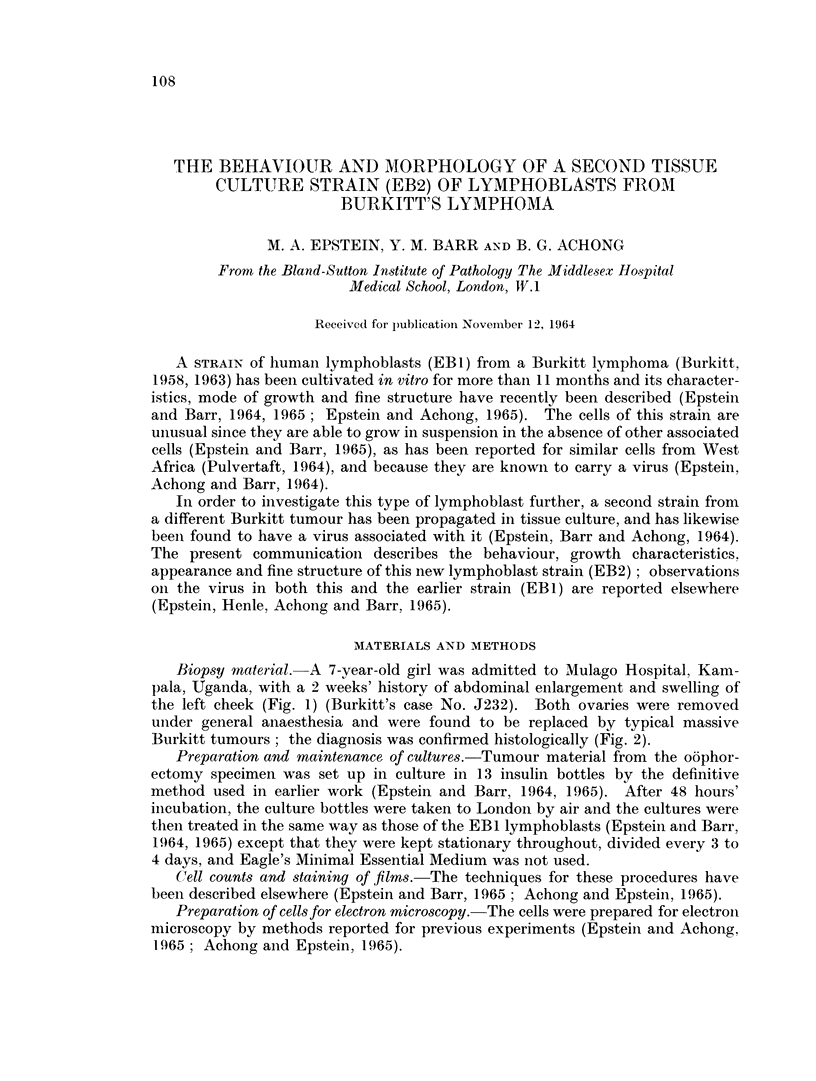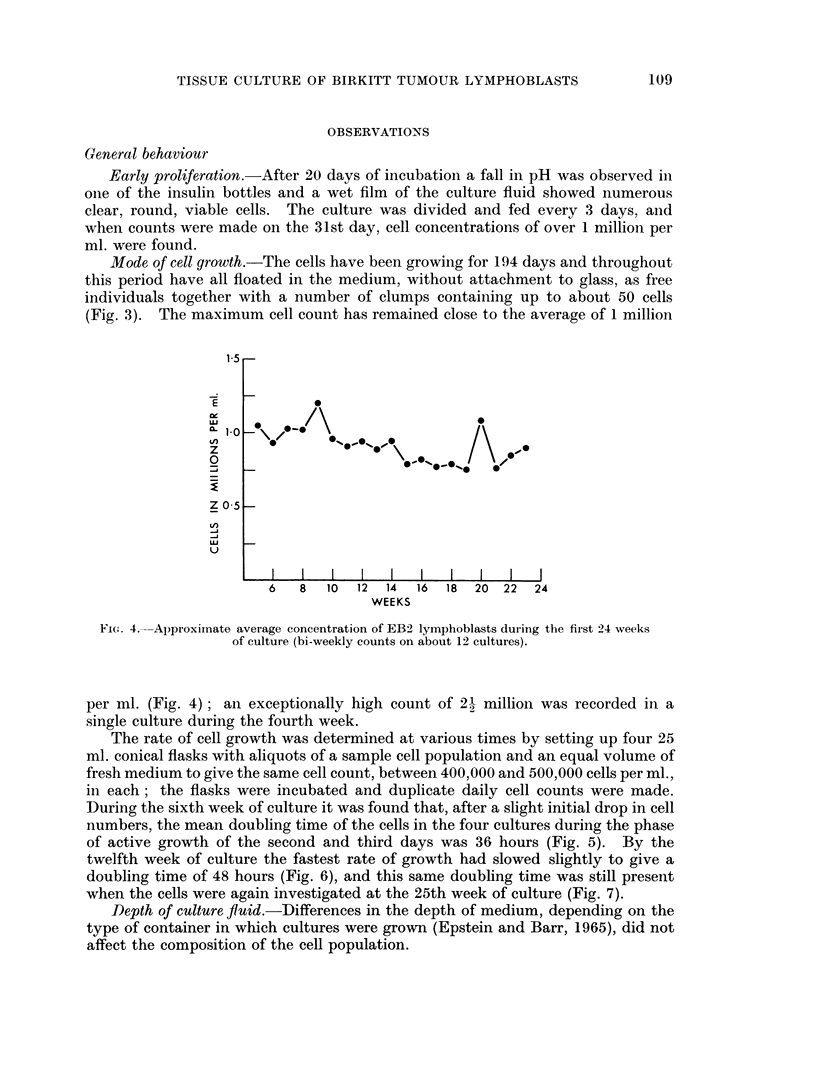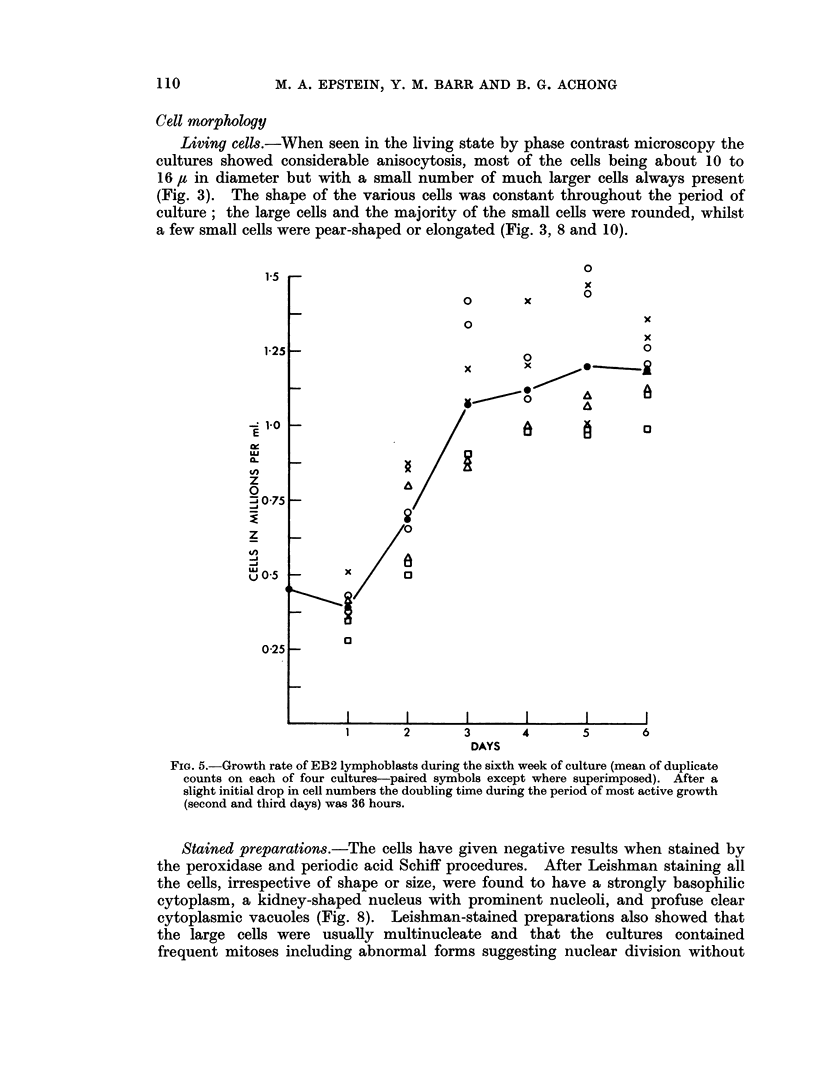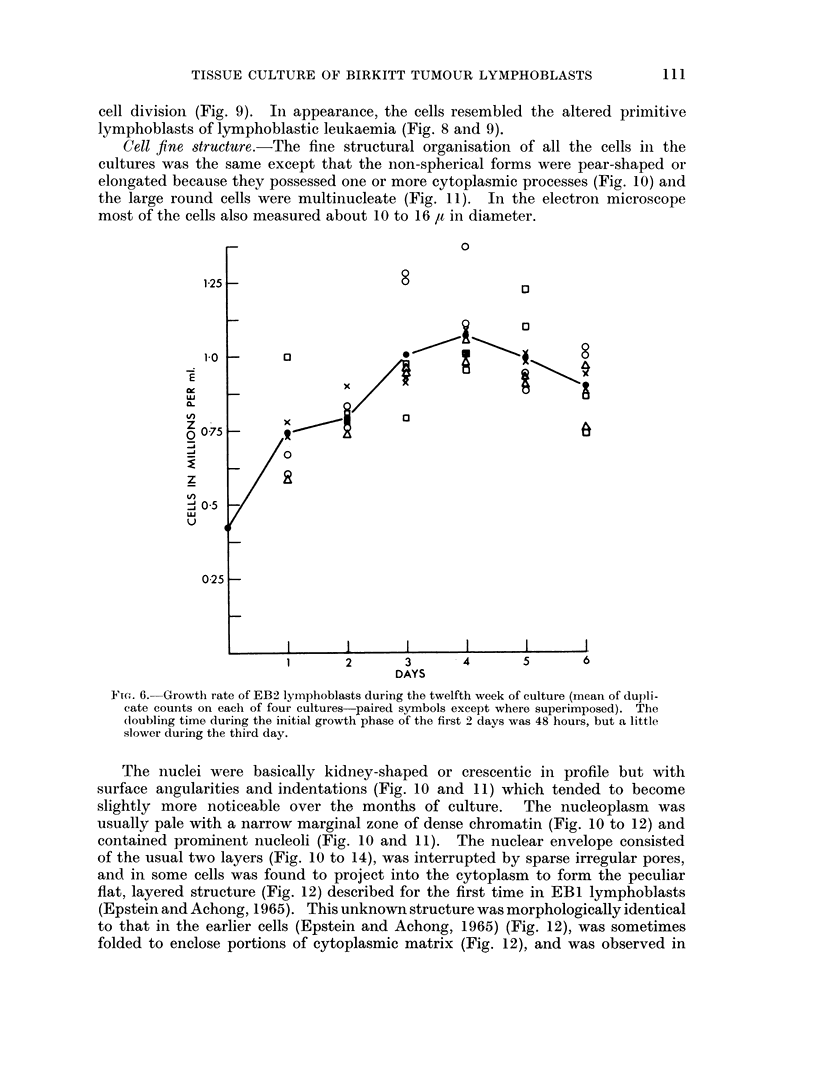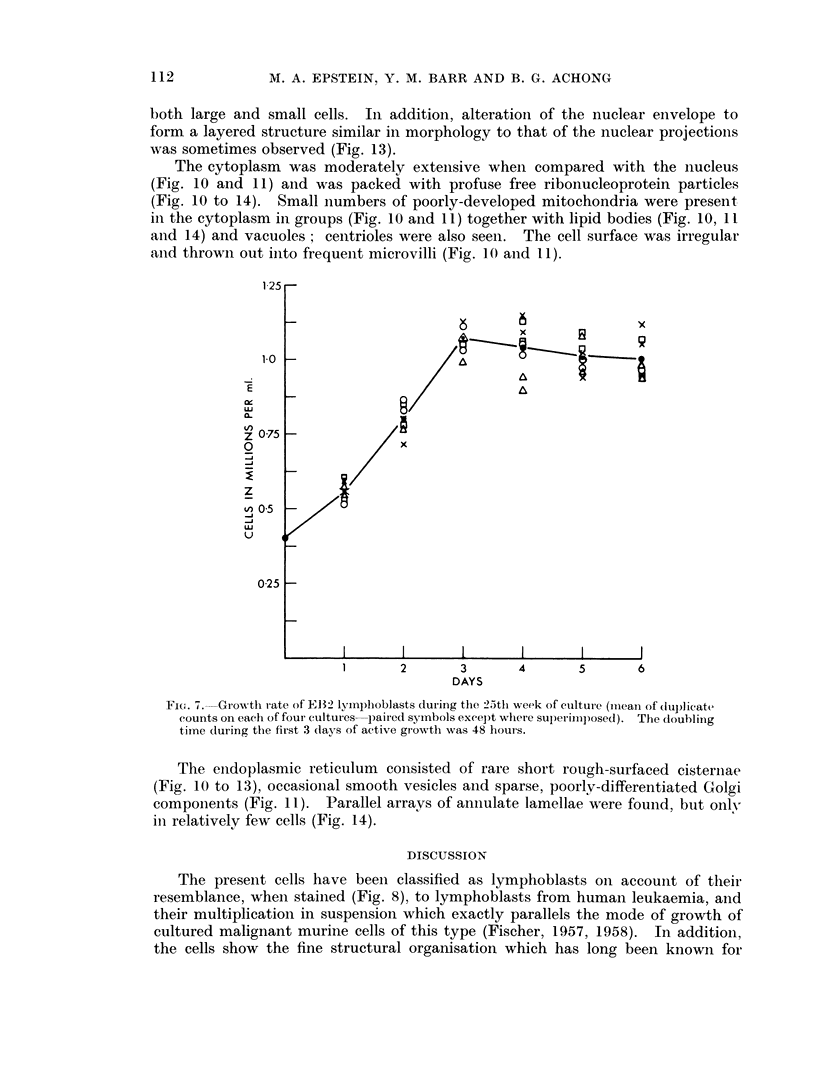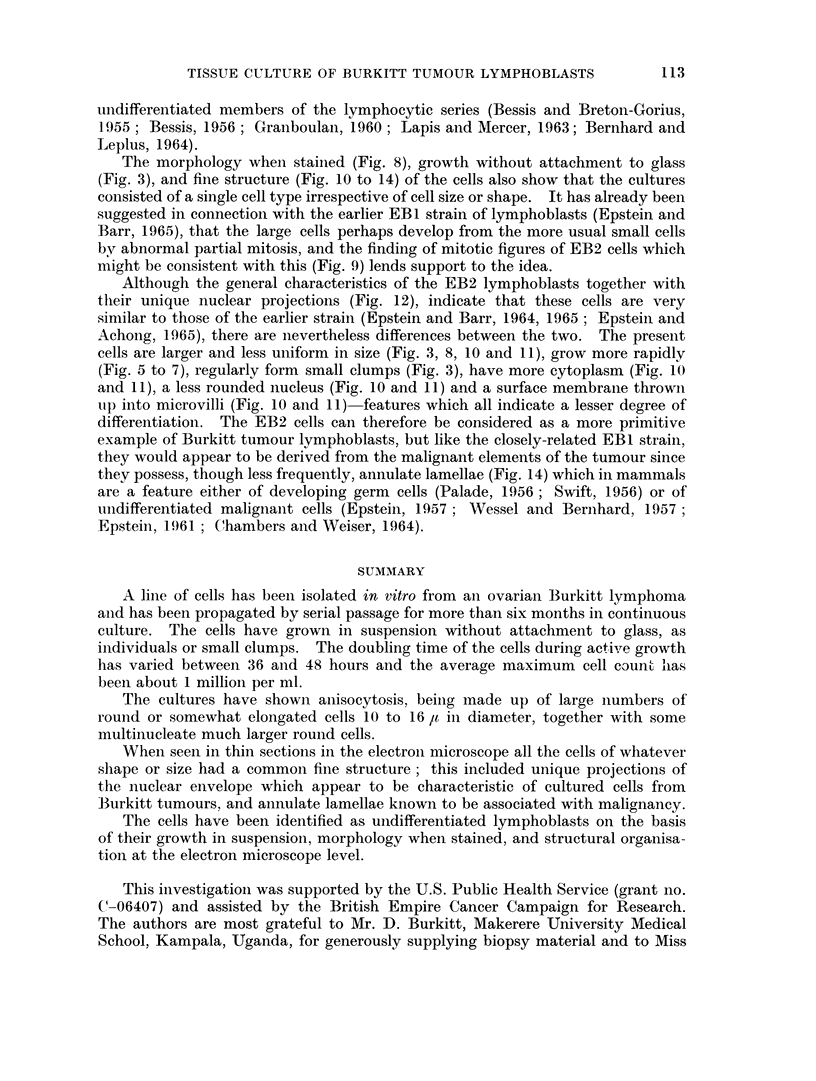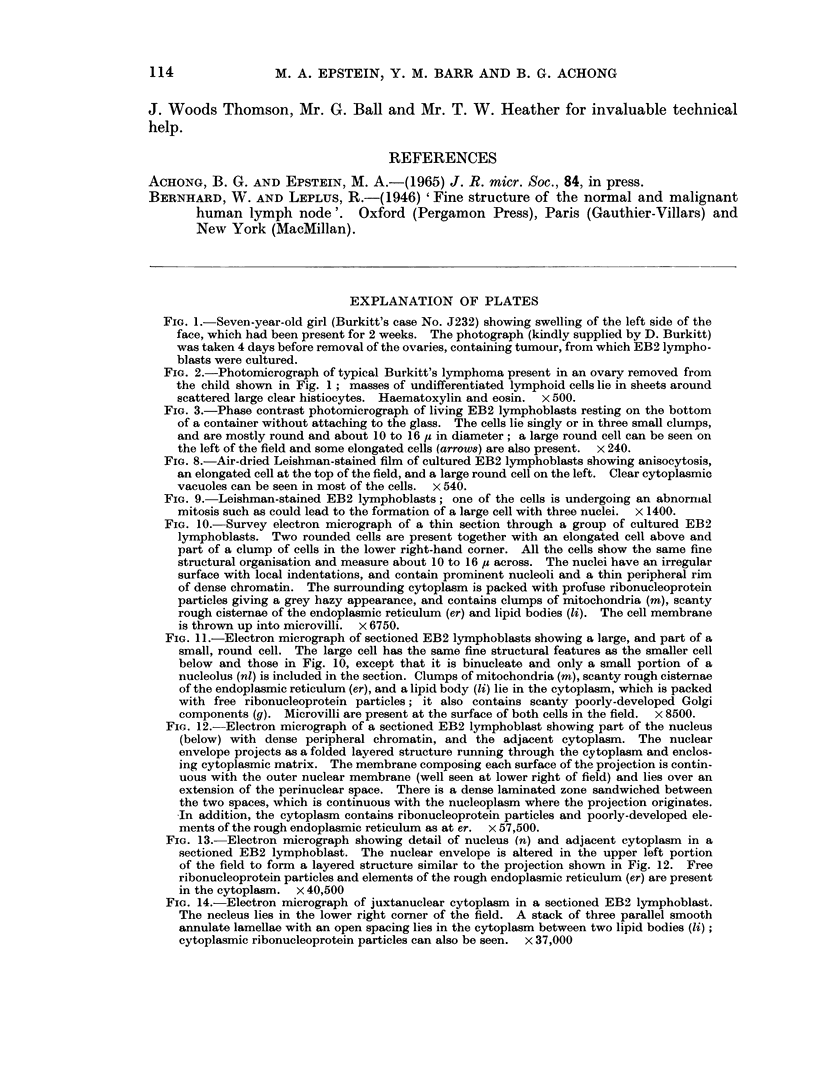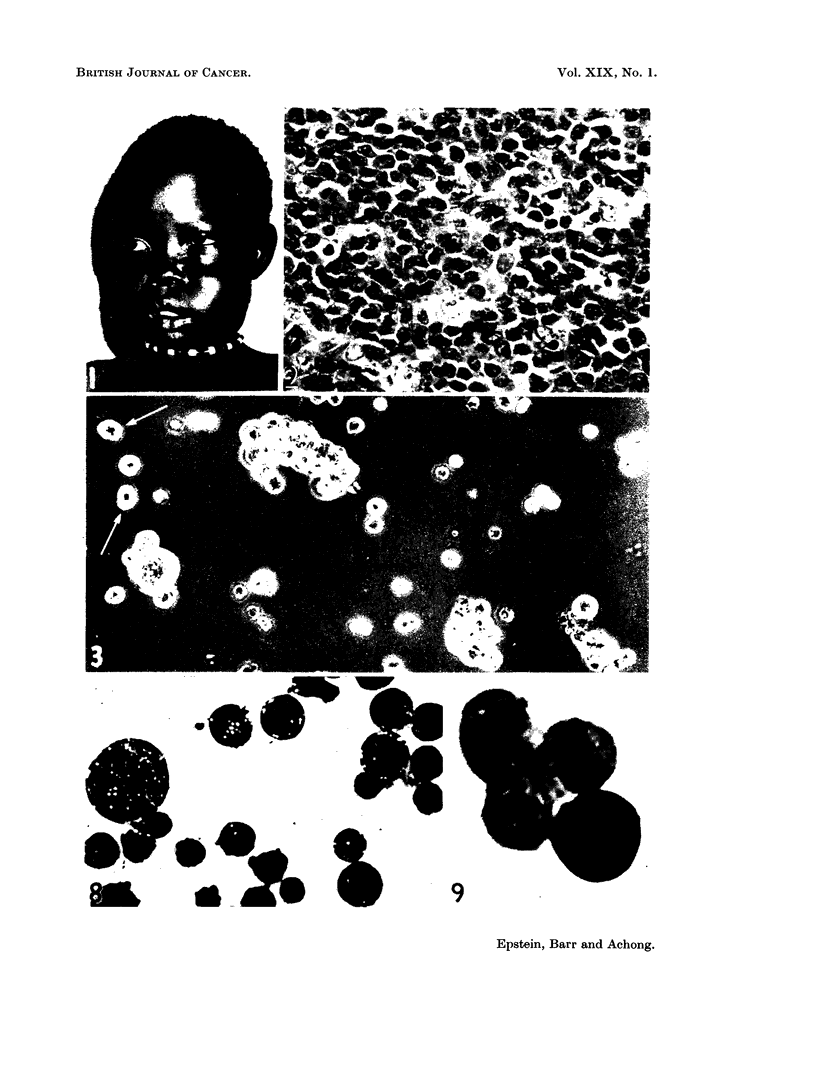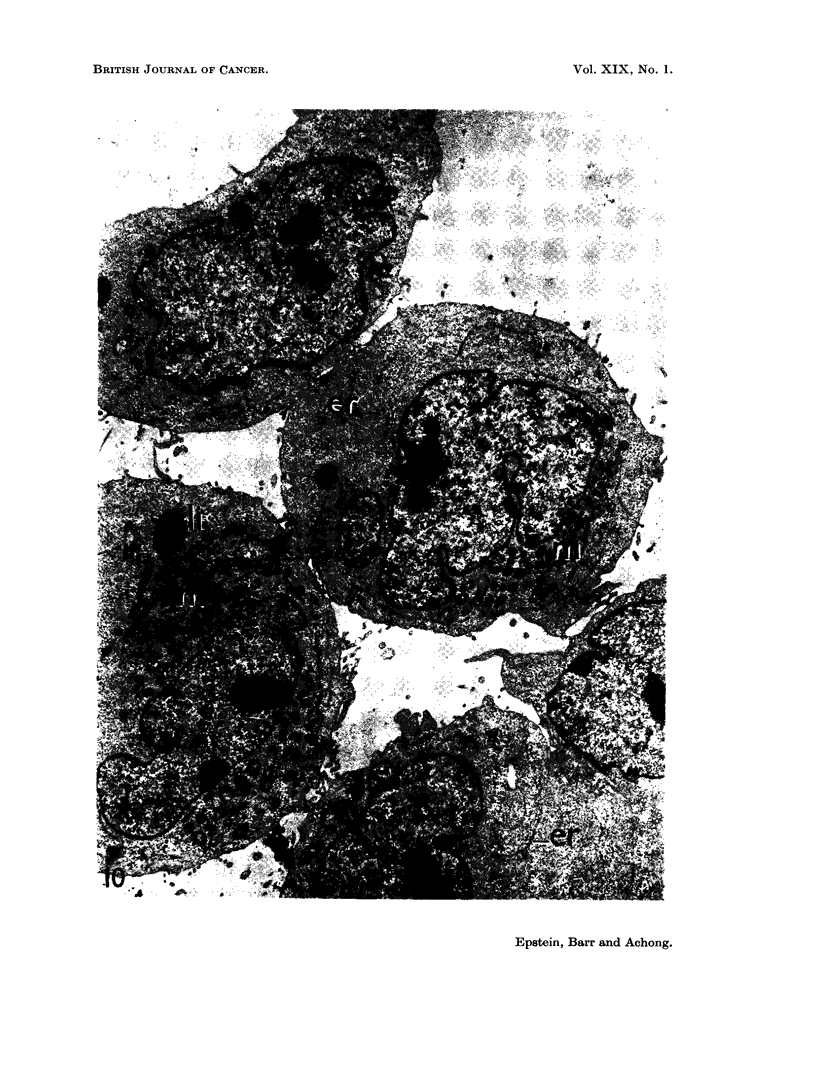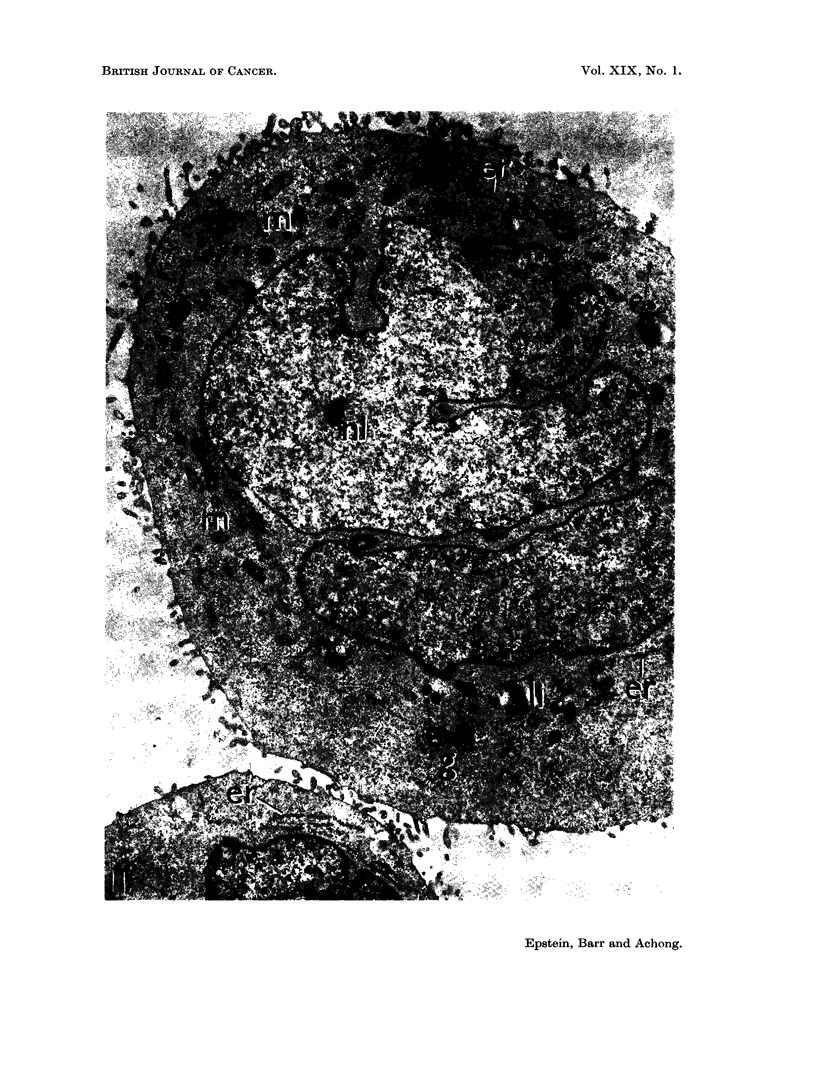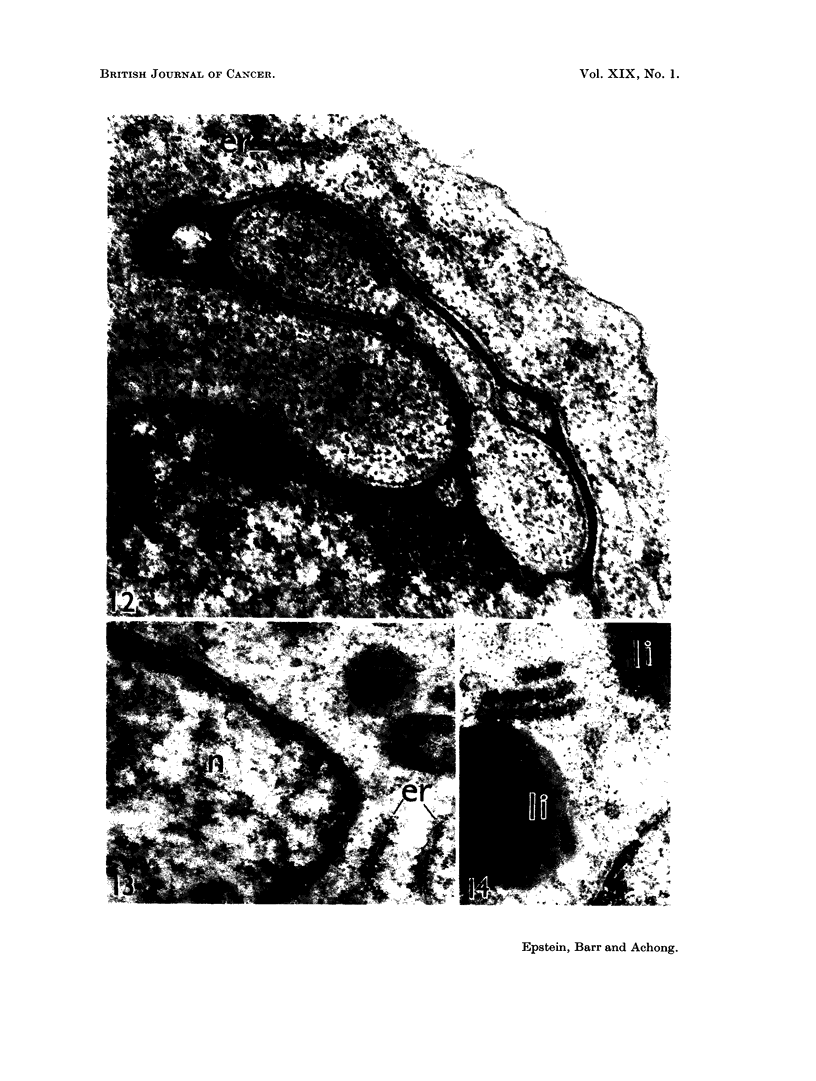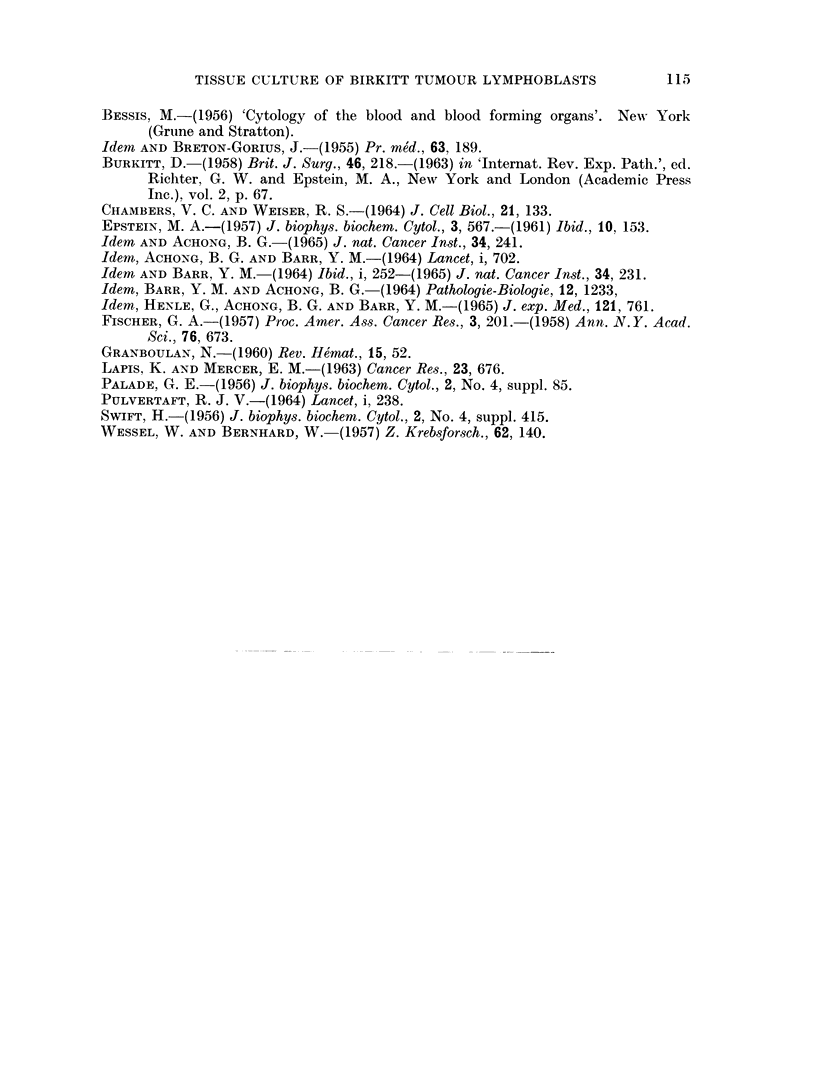# The Behaviour and Morphology of a Second Tissue Culture Strain (EB2) of Lymphoblasts from Burkitt's Lymphoma

**DOI:** 10.1038/bjc.1965.11

**Published:** 1965-03

**Authors:** M. A. Epstein, Y. M. Barr, B. G. Achong

## Abstract

**Images:**


### THE BEHAVIOUR AND MORPHOLOGY OF A SECOND TISSUE CULTURE STRAIN (EB2) OF LYMPHOBLASTS FROM BURKITT'S LYMPHOMA

M. A. EPSTEIN, Y. M. BARR AND B. G. ACHONG

*From the Bland-Sutton Institute of Pathology The Middlesex Hospital Medical School, London, W.1*

Received for publication November 12, 1964

A STRAIN of human lymphoblasts (EB1) from a Burkitt lymphoma (Burkitt, 1958, 1963) has been cultivated *in vitro* for more than 11 months and its characteristics, mode of growth and fine structure have recently been described (Epstein and Barr, 1964, 1965; Epstein and Achong, 1965). The cells of this strain are unusual since they are able to grow in suspension in the absence of other associated cells (Epstein and Barr, 1965), as has been reported for similar cells from West Africa (Pulvertaft, 1964), and because they are known to carry a virus (Epstein, Achong and Barr, 1964).

In order to investigate this type of lymphoblast further, a second strain from a different Burkitt tumour has been propagated in tissue culture, and has likewise been found to have a virus associated with it (Epstein, Barr and Achong, 1964). The present communication describes the behaviour, growth characteristics, appearance and fine structure of this new lymphoblast strain (EB2); observations on the virus in both this and the earlier strain (EB1) are reported elsewhere (Epstein, Henle, Achong and Barr, 1965).

### MATERIALS AND METHODS

*Biopsy material.*—A 7-year-old girl was admitted to Mulago Hospital, Kampala, Uganda, with a 2 weeks' history of abdominal enlargement and swelling of the left cheek (Fig. 1) (Burkitt's case No. J232). Both ovaries were removed under general anaesthesia and were found to be replaced by typical massive Burkitt tumours; the diagnosis was confirmed histologically (Fig. 2).

*Preparation and maintenance of cultures.*—Tumour material from the oöphorectomy specimen was set up in culture in 13 insulin bottles by the definitive method used in earlier work (Epstein and Barr, 1964, 1965). After 48 hours' incubation, the culture bottles were taken to London by air and the cultures were then treated in the same way as those of the EB1 lymphoblasts (Epstein and Barr, 1964, 1965) except that they were kept stationary throughout, divided every 3 to 4 days, and Eagle's Minimal Essential Medium was not used.

*Cell counts and staining of films.*—The techniques for these procedures have been described elsewhere (Epstein and Barr, 1965; Achong and Epstein, 1965).

*Preparation of cells for electron microscopy.*—The cells were prepared for electron microscopy by methods reported for previous experiments (Epstein and Achong, 1965; Achong and Epstein, 1965).

### OBSERVATIONS

### *General behaviour*

*Early proliferation.*—After 20 days of incubation a fall in pH was observed in one of the insulin bottles and a wet film of the culture fluid showed numerous clear, round, viable cells. The culture was divided and fed every 3 days, and when counts were made on the 31st day, cell concentrations of over 1 million per ml. were found.

*Mode of cell growth.*—The cells have been growing for 194 days and throughout this period have all floated in the medium, without attachment to glass, as free individuals together with a number of clumps containing up to about 50 cells (Fig. 3). The maximum cell count has remained close to the average of 1 million

FIG. 4.—Approximate average concentration of EB2 lymphoblasts during the first 24 weeks of culture (bi-weekly counts on about 12 cultures).

per ml. (Fig. 4) ; an exceptionally high count of 2½ million was recorded in a single culture during the fourth week.

The rate of cell growth was determined at various times by setting up four 25 ml. conical flasks with aliquots of a sample cell population and an equal volume of fresh medium to give the same cell count, between 400,000 and 500,000 cells per ml., in each ; the flasks were incubated and duplicate daily cell counts were made. During the sixth week of culture it was found that, after a slight initial drop in cell numbers, the mean doubling time of the cells in the four cultures during the phase of active growth of the second and third days was 36 hours (Fig. 5). By the twelfth week of culture the fastest rate of growth had slowed slightly to give a doubling time of 48 hours (Fig. 6), and this same doubling time was still present when the cells were again investigated at the 25th week of culture (Fig. 7).

*Depth of culture fluid.*—Differences in the depth of medium, depending on the type of container in which cultures were grown (Epstein and Barr, 1965), did not affect the composition of the cell population.

*Cell morphology*

*Living cells.*—When seen in the living state by phase contrast microscopy the cultures showed considerable anisocytosis, most of the cells being about 10 to 16 $\mu$ in diameter but with a small number of much larger cells always present (Fig. 3). The shape of the various cells was constant throughout the period of culture ; the large cells and the majority of the small cells were rounded, whilst a few small cells were pear-shaped or elongated (Fig. 3, 8 and 10).

FIG. 5.—Growth rate of EB2 lymphoblasts during the sixth week of culture (mean of duplicate counts on each of four cultures—paired symbols except where superimposed). After a slight initial drop in cell numbers the doubling time during the period of most active growth (second and third days) was 36 hours.

*Stained preparations.*—The cells have given negative results when stained by the peroxidase and periodic acid Schiff procedures. After Leishman staining all the cells, irrespective of shape or size, were found to have a strongly basophilic cytoplasm, a kidney-shaped nucleus with prominent nucleoli, and profuse clear cytoplasmic vacuoles (Fig. 8). Leishman-stained preparations also showed that the large cells were usually multinucleate and that the cultures contained frequent mitoses including abnormal forms suggesting nuclear division without cell division (Fig. 9). In appearance, the cells resembled the altered primitive lymphoblasts of lymphoblastic leukaemia (Fig. 8 and 9).

*Cell fine structure.*—The fine structural organisation of all the cells in the cultures was the same except that the non-spherical forms were pear-shaped or elongated because they possessed one or more cytoplasmic processes (Fig. 10) and the large round cells were multinucleate (Fig. 11). In the electron microscope most of the cells also measured about 10 to 16 $\mu$ in diameter.

FIG. 6.—Growth rate of EB2 lymphoblasts during the twelfth week of culture (mean of duplicate counts on each of four cultures—paired symbols except where superimposed). The doubling time during the initial growth phase of the first 2 days was 48 hours, but a little slower during the third day.

The nuclei were basically kidney-shaped or crescentic in profile but with surface angularities and indentations (Fig. 10 and 11) which tended to become slightly more noticeable over the months of culture. The nucleoplasm was usually pale with a narrow marginal zone of dense chromatin (Fig. 10 to 12) and contained prominent nucleoli (Fig. 10 and 11). The nuclear envelope consisted of the usual two layers (Fig. 10 to 14), was interrupted by sparse irregular pores, and in some cells was found to project into the cytoplasm to form the peculiar flat, layered structure (Fig. 12) described for the first time in EB1 lymphoblasts (Epstein and Achong, 1965). This unknown structure was morphologically identical to that in the earlier cells (Epstein and Achong, 1965) (Fig. 12), was sometimes folded to enclose portions of cytoplasmic matrix (Fig. 12), and was observed in both large and small cells. In addition, alteration of the nuclear envelope to form a layered structure similar in morphology to that of the nuclear projections was sometimes observed (Fig. 13).

The cytoplasm was moderately extensive when compared with the nucleus (Fig. 10 and 11) and was packed with profuse free ribonucleoprotein particles (Fig. 10 to 14). Small numbers of poorly-developed mitochondria were present in the cytoplasm in groups (Fig. 10 and 11) together with lipid bodies (Fig. 10, 11 and 14) and vacuoles ; centrioles were also seen. The cell surface was irregular and thrown out into frequent microvilli (Fig. 10 and 11).

FIG. 7.—Growth rate of EB2 lymphoblasts during the 25th week of culture (mean of duplicate counts on each of four cultures—paired symbols except where superimposed). The doubling time during the first 3 days of active growth was 48 hours.

The endoplasmic reticulum consisted of rare short rough-surfaced cisternae (Fig. 10 to 13), occasional smooth vesicles and sparse, poorly-differentiated Golgi components (Fig. 11). Parallel arrays of annulate lamellae were found, but only in relatively few cells (Fig. 14).

### DISCUSSION

The present cells have been classified as lymphoblasts on account of their resemblance, when stained (Fig. 8), to lymphoblasts from human leukaemia, and their multiplication in suspension which exactly parallels the mode of growth of cultured malignant murine cells of this type (Fischer, 1957, 1958). In addition, the cells show the fine structural organisation which has long been known for undifferentiated members of the lymphocytic series (Bessis and Breton-Gorius, 1955 ; Bessis, 1956 ; Granboulan, 1960 ; Lapis and Mercer, 1963; Bernhard and Leplus, 1964).

The morphology when stained (Fig. 8), growth without attachment to glass (Fig. 3), and fine structure (Fig. 10 to 14) of the cells also show that the cultures consisted of a single cell type irrespective of cell size or shape. It has already been suggested in connection with the earlier EB1 strain of lymphoblasts (Epstein and Barr, 1965), that the large cells perhaps develop from the more usual small cells by abnormal partial mitosis, and the finding of mitotic figures of EB2 cells which might be consistent with this (Fig. 9) lends support to the idea.

Although the general characteristics of the EB2 lymphoblasts together with their unique nuclear projections (Fig. 12), indicate that these cells are very similar to those of the earlier strain (Epstein and Barr, 1964, 1965 ; Epstein and Achong, 1965), there are nevertheless differences between the two. The present cells are larger and less uniform in size (Fig. 3, 8, 10 and 11), grow more rapidly (Fig. 5 to 7), regularly form small clumps (Fig. 3), have more cytoplasm (Fig. 10 and 11), a less rounded nucleus (Fig. 10 and 11) and a surface membrane thrown up into microvilli (Fig. 10 and 11)—features which all indicate a lesser degree of differentiation. The EB2 cells can therefore be considered as a more primitive example of Burkitt tumour lymphoblasts, but like the closely-related EB1 strain, they would appear to be derived from the malignant elements of the tumour since they possess, though less frequently, annulate lamellae (Fig. 14) which in mammals are a feature either of developing germ cells (Palade, 1956 ; Swift, 1956) or of undifferentiated malignant cells (Epstein, 1957 ; Wessel and Bernhard, 1957 ; Epstein, 1961 ; Chambers and Weiser, 1964).

### SUMMARY

A line of cells has been isolated *in vitro* from an ovarian Burkitt lymphoma and has been propagated by serial passage for more than six months in continuous culture. The cells have grown in suspension without attachment to glass, as individuals or small clumps. The doubling time of the cells during active growth has varied between 36 and 48 hours and the average maximum cell count has been about 1 million per ml.

The cultures have shown anisocytosis, being made up of large numbers of round or somewhat elongated cells 10 to 16 $\mu$ in diameter, together with some multinucleate much larger round cells.

When seen in thin sections in the electron microscope all the cells of whatever shape or size had a common fine structure ; this included unique projections of the nuclear envelope which appear to be characteristic of cultured cells from Burkitt tumours, and annulate lamellae known to be associated with malignancy.

The cells have been identified as undifferentiated lymphoblasts on the basis of their growth in suspension, morphology when stained, and structural organisation at the electron microscope level.

This investigation was supported by the U.S. Public Health Service (grant no. C–06407) and assisted by the British Empire Cancer Campaign for Research. The authors are most grateful to Mr. D. Burkitt, Makerere University Medical School, Kampala, Uganda, for generously supplying biopsy material and to Miss

J. Woods Thomson, Mr. G. Ball and Mr. T. W. Heather for invaluable technical help.

### REFERENCES

ACHONG, B. G. AND EPSTEIN, M. A.—(1965) *J. R. micr. Soc.*, **84**, in press.

BERNHARD, W. AND LEPLUS, R.—(1946) 'Fine structure of the normal and malignant human lymph node'. Oxford (Pergamon Press), Paris (Gauthier-Villars) and New York (MacMillan).

---

### EXPLANATION OF PLATES

FIG. 1.—Seven-year-old girl (Burkitt's case No. J232) showing swelling of the left side of the face, which had been present for 2 weeks. The photograph (kindly supplied by D. Burkitt) was taken 4 days before removal of the ovaries, containing tumour, from which EB2 lymphoblasts were cultured.

FIG. 2.—Photomicrograph of typical Burkitt's lymphoma present in an ovary removed from the child shown in Fig. 1; masses of undifferentiated lymphoid cells lie in sheets around scattered large clear histiocytes. Haematoxylin and eosin. × 500.

FIG. 3.—Phase contrast photomicrograph of living EB2 lymphoblasts resting on the bottom of a container without attaching to the glass. The cells lie singly or in three small clumps, and are mostly round and about 10 to 16 μ in diameter; a large round cell can be seen on the left of the field and some elongated cells (*arrows*) are also present. × 240.

FIG. 8.—Air-dried Leishman-stained film of cultured EB2 lymphoblasts showing anisocytosis, an elongated cell at the top of the field, and a large round cell on the left. Clear cytoplasmic vacuoles can be seen in most of the cells. × 540.

FIG. 9.—Leishman-stained EB2 lymphoblasts; one of the cells is undergoing an abnormal mitosis such as could lead to the formation of a large cell with three nuclei. × 1400.

FIG. 10.—Survey electron micrograph of a thin section through a group of cultured EB2 lymphoblasts. Two rounded cells are present together with an elongated cell above and part of a clump of cells in the lower right-hand corner. All the cells show the same fine structural organisation and measure about 10 to 16 μ across. The nuclei have an irregular surface with local indentations, and contain prominent nucleoli and a thin peripheral rim of dense chromatin. The surrounding cytoplasm is packed with profuse ribonucleoprotein particles giving a grey hazy appearance, and contains clumps of mitochondria (*m*), scanty rough cisternae of the endoplasmic reticulum (*er*) and lipid bodies (*li*). The cell membrane is thrown up into microvilli. × 6750.

FIG. 11.—Electron micrograph of sectioned EB2 lymphoblasts showing a large, and part of a small, round cell. The large cell has the same fine structural features as the smaller cell below and those in Fig. 10, except that it is binucleate and only a small portion of a nucleolus (*nl*) is included in the section. Clumps of mitochondria (*m*), scanty rough cisternae of the endoplasmic reticulum (*er*), and a lipid body (*li*) lie in the cytoplasm, which is packed with free ribonucleoprotein particles; it also contains scanty poorly-developed Golgi components (*g*). Microvilli are present at the surface of both cells in the field. × 8500.

FIG. 12.—Electron micrograph of a sectioned EB2 lymphoblast showing part of the nucleus (below) with dense peripheral chromatin, and the adjacent cytoplasm. The nuclear envelope projects as a folded layered structure running through the cytoplasm and enclosing cytoplasmic matrix. The membrane composing each surface of the projection is continuous with the outer nuclear membrane (well seen at lower right of field) and lies over an extension of the perinuclear space. There is a dense laminated zone sandwiched between the two spaces, which is continuous with the nucleoplasm where the projection originates. In addition, the cytoplasm contains ribonucleoprotein particles and poorly-developed elements of the rough endoplasmic reticulum as at *er*. × 57,500.

FIG. 13.—Electron micrograph showing detail of nucleus (*n*) and adjacent cytoplasm in a sectioned EB2 lymphoblast. The nuclear envelope is altered in the upper left portion of the field to form a layered structure similar to the projection shown in Fig. 12. Free ribonucleoprotein particles and elements of the rough endoplasmic reticulum (*er*) are present in the cytoplasm. × 40,500

FIG. 14.—Electron micrograph of juxtanuclear cytoplasm in a sectioned EB2 lymphoblast. The necleus lies in the lower right corner of the field. A stack of three parallel smooth annulate lamellae with an open spacing lies in the cytoplasm between two lipid bodies (*li*); cytoplasmic ribonucleoprotein particles can also be seen. × 37,000

1

2

3

8

9

Epstein, Barr and Achong.

Epstein, Barr and Achong.

Epstein, Barr and Achong.

BESSIS, M.—(1956) 'Cytology of the blood and blood forming organs'. New York (Grune and Stratton).
*Idem* AND BRETON-GORIUS, J.—(1955) *Pr. méd.*, **63**, 189.
BURKITT, D.—(1958) *Brit. J. Surg.*, **46**, 218.—(1963) *in* 'Internat. Rev. Exp. Path.', ed. Richter, G. W. and Epstein, M. A., New York and London (Academic Press Inc.), vol. 2, p. 67.
CHAMBERS, V. C. AND WEISER, R. S.—(1964) *J. Cell Biol.*, **21**, 133.
EPSTEIN, M. A.—(1957) *J. biophys. biochem. Cytol.*, **3**, 567.—(1961) *Ibid.*, **10**, 153.
*Idem* AND ACHONG, B. G.—(1965) *J. nat. Cancer Inst.*, **34**, 241.
*Idem*, ACHONG, B. G. AND BARR, Y. M.—(1964) *Lancet*, i, 702.
*Idem* AND BARR, Y. M.—(1964) *Ibid.*, i, 252—(1965) *J. nat. Cancer Inst.*, **34**, 231.
*Idem*, BARR, Y. M. AND ACHONG, B. G.—(1964) *Pathologie-Biologie*, **12**, 1233,
*Idem*, HENLE, G., ACHONG, B. G. AND BARR, Y. M.—(1965) *J. exp. Med.*, **121**, 761.
FISCHER, G. A.—(1957) *Proc. Amer. Ass. Cancer Res.*, **3**, 201.—(1958) *Ann. N.Y. Acad. Sci.*, **76**, 673.
GRANBOULAN, N.—(1960) *Rev. Hémat.*, **15**, 52.
LAPIS, K. AND MERCER, E. M.—(1963) *Cancer Res.*, **23**, 676.
PALADE, G. E.—(1956) *J. biophys. biochem. Cytol.*, **2**, No. 4, suppl. 85.
PULVERTAFT, R. J. V.—(1964) *Lancet*, i, 238.
SWIFT, H.—(1956) *J. biophys. biochem. Cytol.*, **2**, No. 4, suppl. 415.
WESSEL, W. AND BERNHARD, W.—(1957) *Z. Krebsforsch.*, **62**, 140.